# Survival and complication rates of two dental implant systems supporting fixed restorations: 10-year data of a randomized controlled clinical study

**DOI:** 10.1007/s00784-023-05323-5

**Published:** 2023-10-31

**Authors:** Naida Gadzo, Alexis Ioannidis, Nadja Naenni, Jürg Hüsler, Ronald E. Jung, Daniel S. Thoma

**Affiliations:** 1 Clinic of Reconstructive Dentistry, Center for Dental Medicine, University of Zurich, 8032 Zurich, CH Switzerland; 2 Department of Periodontology, Research Institute for Periodontal Regeneration, College of Dentistry, Yonsei University, Seoul, Korea

**Keywords:** Dental implants, Marginal bone level, Technical complications, Biological complications, Survival, Long-term

## Abstract

**Objectives:**

To compare clinical, radiographic, biological and technical long-term outcomes of two types of dental implants over a period of 10 years.

**Materials and methods:**

Ninety-eight implants were placed in 64 patients**,** randomly allocated to one of two manufacturers (AST and STM). All implants were loaded with fixed restorations. Outcome measures were assessed at implant insertion (T_i_), at baseline examination (T_L_), at 1, 3, 5, 8 and 10 (T_10_) years. Data analysis included survival, bone level changes, complications and clinical measures.

**Results:**

Re-examination was performed in 43 patients (23 AST and 20 STM) at 10 years. The implant level analysis was based on 37 (AST) and 32 (STM) implants. Survival rates of 100% were obtained for both groups. The median changes of the marginal bone levels between baseline and T_10_ (the primary endpoint) amounted to a loss of 0.07 mm for group AST and a gain of 0.37 mm for group STM (intergroup *p =* 0.008).

Technical complications occurred in 27.0% of the implants in group AST and in 15.6% in group STM.

The prevalence of peri-implant mucositis was 29.7% (AST) and 50.1% (STM). The prevalence of peri-implantitis amounted to 0% (AST) and 6.3% (STM).

**Conclusions:**

Irrespective of the implant system used, the survival rates after 10 years were high. Minimal bone level changes were observed, statistically significant but clinically negligible in favor of STM. Technical complications were more frequently encountered in group AST, while group STM had a higher prevalence of peri-implant mucositis.

## Introduction

Implant dentistry has substantially changed the way patients are treated with fixed and removable prosthetic solutions to reestablish chewing function and esthetics [1–7]. Three decades ago, dental implants were predominantly placed by specialists and at university settings as demonstrated by systematic reviews. Over time, implant dentistry has shifted to general practitioners and subsequently resulted in a highly increasing number of implants placed per year [8]. Originally, dental implants were considered a therapeutic option in edentulous patients. Improvements made in terms of design, surface characteristics, materials, prosthetic components further expanded treatment options using dental implants. According to industry reports, the worldwide market for dental implants is valued at approximately 12–18 million implants sold per year, with over 100 distinct commercial brands providing a range of implant options [9]. Consequently, clinicians are facing difficulties to make a choice for a specific implant system. From a scientific point of view, a number of parameters is considered crucial in the decision-making process for a specific manufacturer and implant system. This includes scientifically documented clinical and radiographic long-term outcome measures and the reporting of technical, biological and esthetic complications, survival rates and implant failures [10]. Based on the success criteria defined in the early eighties, implant success rates consist of the establishment of an osseointegration of the implant, no evidence of peri-implant radiolucency, a mean bone loss of less than 0.2 mm annually after the first year of service, no pain or discomfort attributed to the implant, no preclusion of a placement of a crown with a satisfactory appearance and a minimum success rate of 85% after 5 years and 80% at the end of a 10-year period [11, 12]. The implant design currently preferred by clinicians is a two-piece implant with a non-matching implant abutment junction. Preclinical and clinical studies demonstrated favorable data in terms of the maintenance of marginal bone [4, 5, 13, 14].

These data indicate that the proposed success criteria are being complied up to the present time. Apart from the traditional measurement of marginal bone levels and implant survival rates, long-term controlled studies are needed to report on the rate of technical and biological complications. This is of even more importance since substantial changes were made on the level of the prosthetic restorations. Veneered ceramic implant-supported single crowns showed significantly higher rates for ceramic chipping compared to monolithic ceramics [15]. The implant-crown transition zone, especially in the esthetic region has gained interest, as a greater stability of the mucosal margin could be shown when using implant provisionals with a concave compared to a convex profile [16].

Therefore, the aim of the present study was to assess clinical, technical, biological and radiographic long-term outcomes of two types of dental implants at 10 years. Both analyzed implant types are characterized by a non-matching implant-abutment junction and support fixed restorations.

## Material and methods

### Study design

The present randomized controlled clinical trial was approved by the local ethics committee (Kantonale Ethikkommission Kanton Zürich, Ref. Nr. KEK-ZH-Nr. 2013–0121) and was performed following the principles outlined in the World's Medical Association's Declaration of Helsinki on experimentation involving human subjects (“World Medical Association Declaration of Helsinki: ethical principles for medical research involving human subjects.”, 2013).

Sixty-four patients were consecutively enrolled at the Clinic of Reconstructive Dentistry, University of Zurich, Switzerland, after signing the informed consent. All treated patients were in need of dental implant therapy with fixed dental restorations. Using a computer-generated randomization, enrolled patients were randomly assigned to receive dental implants from either one of two manufacturers: AST (OsseoSpeed TX 3.0–5.0 S, TX 4.5; Astra Tech Implant System, Dentsply Sirona) or STM (Straumann Bone Level Implants 3.3, 4.1, 4.8 mm, SLAactive; Straumann AG). The inclusion and exclusion criteria, the specific surgical procedure as well as the prosthetic protocol, were already reported in previously published literature [17, 18].

In summary, enrolled patients were healthy and of legal age, showed neither local oral nor systemic pathologies, performed good oral hygiene (full mouth plaque control record < 25% [19]) with adequate control of inflammation (full mouth bleeding on probing < 25% [20]) and were in need of implant therapy with fixed restorations. The location of the implants was not limited to the upper/lower jaw or anterior/posterior sites, nor were implants in need of bone regeneration excluded.

The surgical procedures followed the manufacturers’ recommendations as well as the standard protocol of the clinic. A standardization of the vertical implant position was not pursued, given the necessity to account for influential factors such as mucosal thickness and the prosthetic restoration design. This approach acknowledges the individualized nature of implant placement, where optimizing the vertical position requires careful consideration of these variables. Implants requiring a guided bone regeneration (GBR), in cases of a dehiscence or a fenestration defect, were grafted with demineralized bovine bone mineral (DBBM) (Bio-Oss Spongiosa; Geistlich Pharma AG) and a resorbable (Bio-Gide, Geistlich Pharma AG) or a non-resorbable (Gore-Tex; W.L. Gore & Assoc.) membrane to cover the bone substitute. Depending on the surgeon’s preference and the clinical situation, in some cases synthetic bone grafting materials were applied.

The prosthetic treatments were performed according to the guidelines of the respective implant systems. Implant restorations were screw-retained or cemented based on the clinical situation and the clinician’s preference.

Baseline was defined as the timepoint of the insertion of the final restoration. The maintenance care with a regular recall including dental hygiene sessions was personalized for every patient at the baseline appointment. Follow-up examinations were scheduled at 1, 3, 5, 8 and 10 years after baseline. This article is reported in compliance with the according CONSORT guidelines for the examination of parallel group randomized trials.

The primary outcome of the study was marginal bone level changes. Secondary outcomes were implant and restoration survival rates, clinical, biological and technical outcomes.

### Outcome measures

Six sites per implant (mesiobuccal, buccal, distobuccal, distolingual, lingual and mesiolingual) and the neighboring teeth/implant(s) and contralateral tooth or implant sites were assessed for clinical measurements using a periodontal probe (UNC-15, Hu-Friedy, Chicago, IL, USA) at each follow-up appointment. All measurements were performed by examiners not involved into the clinical therapy. One single examiner performed the 10-year follow-up examinations, after a calibration process for clinial trials at the Clinic of Reconstructive Dentistry, University of Zurich. The variables examined are listed as follows:

- Probing depth (PD, mm)
- Bleeding on probing (BOP, %) [20]
- Plaque control record (PCR, %) [19]

Outcome measures were assessed at implant insertion (T_i_), at the baseline examination (insertion of final restoration; T_L_), at 1, 3, 5, 8 and 10 (T_10_) years after loading/baseline. Data for 1, 3, 5 and 8 years are not reported in the present manuscript.

Standardized periapical radiographs of all implants were taken using a paralleling technique with Rinn-holders at T_i_ and all the follow-up time-points. After conversion to.jpeg files and importing them in an open-source software (Image J; National Institutes of Health, Bethesda, MD, USA), marginal bone level (MBL) changes were measured at a magnification of 10-15x. For the calibration and determination of the exact magnification of the files, the pitch distance between two implant threads was taken as a reference. The marginal bone level (MBL) was recorded by measuring the distance from the flat top of the implant shoulder to the bone crest using a scale divided into 0.1 mm steps at two sites, the mesial and distal implant surface of each dental implant (distance implant bone, DIB). The differences between the time-points were then used to calculate MBL changes. Radiographic measurements were performed by one single examiner, who underwent comprehensive training by senior faculties to ensure consistency in evaluations. To enhance the reliability of our findings, a random subset of samples was assessed independently by another examiner.

### Biological complications

Peri‐implant mucositis and periimplantitis were defined according to the consensus report of the 2017 World Workshop on the Classification of Periodontal and Peri‐Implant Diseases and Conditions [21, 22].

The incidence of biological complications was assessed at the follow-up visits or in case patients came in for an extra visit.

Peri-implant health was defined as absence of erythema, bleeding on probing, swelling and suppuration. Biological complications included peri-implant mucositis (BOP +) and peri-implantitis (BOP + , marginal bone loss beyond the initial bone remodelling (> 0.5 mm); progressive marginal bone loss between 1 and 10 years).

### Technical complications

The occurrence of technical complications was registered once per time-point for each implant. Technical complications encompassed: chippings, screw loosening and screw fracture, abutment fracture and the loss of the implant crown.

### Statistical analysis

Data were collected and added to a spreadsheet (Microsoft Excel, Microsoft Corporation, Redmond, Washington, USA). Statistical analysis was performed with a statistical analysis software (SAS 9.4, SAS Corp., Cary NC. USA). Descriptive summary statistics was obtained on the implant level as well on the patient level.

On the patient level intragroup comparisons for the medians of the groups were performed by Wilcoxon-signed-rank test and the intergroup comparisons of the two groups were based on the Wilcoxon-Mann–Whitney test. Hodges-Lehmann 95%-confidence estimations are applied for the medians or for the difference of the medians.

If the normality assumption was holding, F-tests were applied and 95%-confidence intervals for the means or differences of the means were determined.

Nominal data of the two groups were compared on the patient level with the chi-squares test with exact derivations of the p-values.

For the implant level mixed linear models were applied for the comparisons because of the dependence of the data within a patient.

The level of significance was set at 5%. No correction of the multiple testing is applied.

For the analyses of further impact factors we applied linear models for the patient level data and linear mixed models for the implant level data.

## Results

### Demographic data

Ninety-eight implants (31 in the mandible and 67 in the maxilla) were placed in 64 enrolled patients, eventually receiving fixed restorations at the Clinic of Reconstructive Dentistry, University of Zurich, between February and December 2009.

Re-examination was performed in 43 patients with a mean age of 67.3 years (SD ± 11.0) at 10 years (mean 10.4y). The drop-out rate was 31.25%. Reasons for loss of follow-up were: moving further away or abroad, decreased mobility because of age or general health conditions and passing away.

The implant level analysis was based on 37 (AST) and 32 (STM) implants at T_10_. One implant was randomly selected (by the statistician) in every patient serving as a basis for the patient level analysis. This encompassed 23 (AST) and 21 (STM) implants at T_10_. Table 1 summarizes baseline and 10-year characteristics of the study cohort, as well as the types of restoration.

**Table 1:** Characteristics of the study cohort at baseline and 10 years, including type of restoration on the implant and patient level for both implant systems (AST and STM)

	Baseline		10 years	
	AST	STM	AST	STM
Age (years)	55 ± 11.6	54.3 ± 16.1	66 ± 10.6	68 ± 12.9
Gender (female, F; male, M)	17F / 16M	21F / 10M	9F / 14M	12F / 8M
Number of patients	33	31	23	21
Number of implants	54	44	37	32
Number of implants upper jaw	35	32	22	22
Number of implants lower jaw	19	12	15	10

	Implant level		Patient level		Implant level		Patient level	
	AST (S1)	STM (S2)	AST (S1)	STM (S2)	AST (S1)	STM (S2)	AST (S1)	STM (S2)
Single crown	29	13	19	12	23	9	14	8
Splinted single crowns	4	2	2	0	2	2	1	0
Multi-unit restorations	13	15	6	8	9	12	5	6
Restorations with cantilevers	8	13	6	11	3	9	3	7
Total Number	54	43	33	31	37	32	23	21

*AST* Astra; *STM*, Straumann

### Radiographic data (primary endpoint)

Table 2aa (implant level analysis) and 2bb (patient level analysis) display all radiographic data. Positive values indicate the implant shoulder to be located more coronally relative to the bone crest.

**Table 2a:** Implant level. Radiographic data of marginal bone levels at the time of insertion (Ti), at loading (TL) and at the 10-year (T10) follow-up examination, including changes between different time-points. Implant level analysis for both implant systems (AST and STM), *p*-values based on mixed model analyses

		AST							STM						
	*n*	Mean (sd) (mm)	Q1	Median (mm)	Q3	Range (mm) min to max	Intra-group *p*-value	*n*	Mean (sd) (mm)	Q1	Median (mm)	Q3	Range (mm) min to max	Intra-group *p*-value	Inter-group *p*- value
Ti	35	−0.98 (0.94)	−1.52	−0.93	−0.35	−4.01 to 0.59	< 0.001	31	−0.98 (1.12)	−1.47	−0.86	−0.06	−4.92 to 0.65	< 0.001	0.425
TL	35	−0.10 (0.40)	−0.36	−0.21	0.18	−0.83 to 1.16	0.287	31	0.04 (0.54)	−0.10	0.09	0.36	−1.70 to 1.10	0.611	0.273
T10	35	−0.10 (0.47)	−0.40	−0.20	0.11	−1.29 to 1.03	0.577	31	−0.78 (0.96)	−0.90	−0.47	−0.20	−4.42 to 0.06	< 0.001	< 0.001
TL-Ti	34	0.86 (1.16)	0.132	0.739	1.40	−1.14 to 5.17	0.011	31	1.02 (1.27)	0.44	0.85	1.50	−2.30 to 4.92	< 0.001	0.042
T10-TL	33	0.01 (0.41)	−0.19	0.03	0.22	−1.09 to 0.98	0.514	31	−0.82 (1.26)	−0.99	−0.42	−0.05	−5.52 to 0.48	< 0.001	< 0.001

*AST*, Astra; *ST*M, Straumann; *SD*, standard deviation; *Q1*, first quartile; *Q3*, third quartile; *Ti*, time of insertion; *TL* timepoint at loading; *T10*, timepoint 10-years

Positive values indicate the implant shoulder to be located more coronally relative to the bone crest

**Table 2b:** Patient level. Radiographic data of marginal bone levels at the time of insertion (Ti), at loading (TL) and at the 10-year (T10) follow-up examination, including changes between different timepoints. Corresponding analysis on patient level for both implant systems (AST and STM). Note: Calculations of p-values for the patient level analysis were performed with Wilcoxon signed rank test (intragroup) and Wilcoxon test (intergroup) to assess their influence of the group or of the time

		AST							STM						
	*n*	Mean ± SD (mm)	Q1	Median (mm)	Q3	Range (mm) min to max	Intra-group *p*-value	*n*	Mean ± SD (mm)	Q1	Median (mm)	Q3	Range (mm) min to max	Intra- group *p*-value	Inter-group *p*- value
Ti	22	−1.25 ± 1.03	−1.79	−1.27	−0.66	−4.01 to 0.59	< 0.001	20	−1.00 ± 1.30	−1.59	−0.85	−0.03	−4.92 to 0.65	0.001	0.252
TL	22	−0.15 ± 0.43	−0.37	−0.31	0.14	−0.83 to 1.16	0.060	20	−0.03 ± 0.60	−0.14	0.08	0.25	−1.71 to 1.10	0.651	0.085
T10	23	−0.11 ± 0.51	−0.41	−0.22	0	−0.83 to 1.29	0.103	20	−0.76 ± 1.05	−0.83	−0.48	−0.10	−4.42 to 0.06	< 0.001	0.042
TL-Ti	22	1.10 ± 1.35	0.483	1.12	1.56	−1.42 to 5.174	< 0.001	20	0.97 ± 1.47	0.37	0.82	1.61	−2.30 to 4.92	0.004	0.659
T10-TL	22	0.06 ± 0.32	−0.18	0.07	0.25	−0.49 to 0.87	0.458	20	−0.73 ± 1.37	−0.80	−0.37	−0.00	−5.52 to 0.48	0.006	0.008

*AST*, Astra; *STM*, Straumann; *SD*, standard deviation; *Q1*, first quartile; *Q3*, third quartile; *Ti*, time of insertion; *T*L timepoint at loading; *T10*, timepoint 10-years

Positive values indicate the implant shoulder to be located more coronally relative to the bone crest

On the patient level, at T_10_, the median relative distances between the implant shoulder and the bone crest were -0.22 mm (Q1: -0.41 mm; Q3: 0.00 mm) in group AST and -0.48 mm (Q1: -0.83; Q3: -0.1 mm) in group STM (intergroup comparison *p =* 0.042 with a 95%- confidence interval (0.000, 0.745) for the difference of the medians of group 2 vs. group 1.).

On the patient level, the median changes of the marginal bone levels between baseline (T_L_) and T_10_ (the primary endpoint) amounted to a loss of 0.07 mm (Q1: -0.18 mm, Q3: 0.25) for group AST, (intragroup *p =* 0.007, 95% CI for the median change: (-0.11, 0.19)) and to a gain of 0.37 mm (Q1: -0.80; Q3: 0.00) for group STM (intragroup *p =* 0.006, 95% CI for the median change: (-1.10, -0.11)) (intergroup comparison test: *p =* 0.008, 95%-confidence interval for median difference between AST and STM: (-0.80, -0.11)). Additional models were fitted and adjusted for type of reconstruction, site (anterior/posterior) and gender and tested for possible interactions. None of the factors revealed a significant influence (*p* > 0.15).

On the implant level analysis for both implant systems (AST and STM), the mean relative distances between the implant shoulder and the bone crest at T_10_ is -0.10 (std: 0.474) for group AST (intragroup *p =* 0.328, 95%-CI (-0.45, 0.16)) and -0.78 (std: 0.960) for group STM (intragroup *p < *0.001, 95%-CI (-1.09, -0.43)), (intergroup *p =* 0.001, 95%-CI (0.16, 1.06)) based on the mixed model analyses.

The mean changes of the marginal bone levels between baseline (T_L_) and T_10_ (the primary endpoint) amounted on the implant level to 0.01 (std: 0.41) for group AST (intragroup *p =* 0.514, 95%-CI (-0.24, 0.46)) and -0.82 (std: 1.26) AST (intragroup *p < *0.001, 95%-CI (-1.07, -0.50), intergroup *p < *0.001, 95%-CI (0.45, 1.35)) based on the mixed model analyses. Additional models were fitted and adjusted for type of reconstruction, site (anterior/posterior) and gender and tested for possible interactions. None of the factors revealed a significant influence (*p* > 0.25).

### Survival rates

During the 10-year follow-up, five implants were lost. In group AST, four implants in three patients were lost due to periimplantitis. In group STM, one implant was lost due to peri-implantitis. This amounts to a survival rate of 89.7% for group AST and 96.8% for group STM. On the patient level with one randomly selected implant contributing to the analysis, survival rates of 100% were obtained in both groups (*p =* 1.00).

### Technical and biological complications on the implant level

Technical complications occurred in 10 out of 37 implants (27.0%) in group AST and in 5 out of 32 implants (15.6%) in group STM during 10 years (intergroup comparison: *p =* 0.261). Within the last two years (8–10 years of follow-up), the technical complication rates were 8.1% (3 of 37 implants) (AST) and 0% (0 of 32 implants) (STM). The most prevalent complications were minor chippings and screw loosening, which were treated chairside. One abutment fractured in group AST.

The prevalence of peri-implant mucositis was 29.7% in group AST (affecting 11 implants) and 50.1% in group STM (16 implants) at T_10_. Additionally, the prevalence of peri-implantitis was 0.0% in group AST (affecting none implant) and 6.3% in group STM (2 implants) at T_10_.

### Technical and biological complications on the patient level

On the patient level, technical complications occurred in 5 out of 23 implants (21.7%) in group AST during 10 years. Within the last two years (8–10 years of follow-up), the technical complication rates were 8.7% (2 of 23 implants). None of the implants in group STM showed a technical complication (0.0%) in the last follow-up visit T_10_, while 3 of 21 implants (14.3%) in group STM presented minor chippings and a loss of the composite seal during the observation period of 10 years overall (intergroup comparison *p =* 0.701).

The prevalence of peri-implant mucositis was 34.8% (8 of 23 implants) in group AST and 52.3% 11 of 21 implants) in group STM at T_10_. The respective prevalence of peri-implantitis amounted to 0% in group AST and 4.8% (affecting 1 implant) in group STM at T_10_.

### Clinical outcome measures

Clinical outcomes on patient level for both implant systems (AST and STM) at the time of loading (TL), and at the 10-year (T_10_) follow-up examination with the respective changes over time are presented in Table 3.

**Table 3:** Clinical outcomes on patient level for both implant systems (AST and STM) at the time of loading (TL), and at the 10-year (T10) follow-up examination with the respective changes over time. Patient level analysis with means, standard deviations (SD), medians, first and third quartile (Q1, Q3), range from minimum to maximum for both implant systems. BOP: bleeding on probing; PCR: plaque control record; PD: probing depth. Calculations of p- values for the patient level analysis were performed with the Wilcoxon signed rank test for the intragroup comparison and the Wilcoxon test for the intergroup comparison

	AST					STM									
	Mean ± SD (mm)	Q1	Median (mm)	Q3	Range (mm) min to max	Intra-group *ρ*-value		Mean ± SD (mm)	Q1	Median **(**mm**)**	Q3	Range (mm) min to max	Intra-group *p*-value	Inter-group *ρ*- value	
PD															
TL	3.1 ± 0.5	3.0	3.2	3.3	1.7 to 4.2	< 0.001		2.7 ± 1.0	2.4	2.7	3.3	0.0 to 4.3	< 0.001		0.092
T10	2.8 ± 0.5	2.5	2.8	3.0	1.8 to 4.0	< 0.001		2.9 ± 0.7	2.6	2.7	3.2	2.0 to 4.5	< 0.001		0.912
T10-TL	−0.3 ± 0.6	−0.7	−0.2	0	−1.5 to 0.8	0.019		0.2 ± 1.1	-0.4	0.2	0.8	−1.8 to 2.7	0.602		0.070
BOP															
TL	0.2 ± 0.2	0.0	0.2	0.5	0.0 to 0.7	< 0.001		0.2 ± 0.2	0.0	0.2	0.3	0.0 to 0.5	< 0.001		0.469
T10	0.1 ± 0.1	0.0	0.0	0.2	0.0 to 0.3	0.008		0.1 ± 0.1	0.0	0.2	0.2	0.0 to 0.5	0.001		0.255
T10-TL	−0.2 ± 0.3	−0.3	−0.2	0	−0.7 to 0.3	0.014		-0.1 ± 0.2	-0.2	-0.2	0.1	−0.3 to 0.3	0.471		0.163
PCR															
TL	0.1 ± 0.1	0.0	0.0	0.0	0.0 to 0.5	0.063		0.1 ± 0.2	0.0	0.0	0.1	0.0 to 0.7	0.063		0.645
T10	0.1 ± 0.1	0.0	0.0	0.2	0.0 to 0.5	0.002		0.2 ± 0.2	0.0	0.0	0.2	0.0 to 1.0	0.008		0.890
T10-TL	0.0 ± 0.2	0	0	0.2	−0.5 to 0.5	0.059		0.3 ± 0.3	0	0	0.2	−0.7 to 0.8	0.385		0.639

Abbreviations: AST, Astra; STM, Straumann; SD, standard deviation; Q1, first quartile; Q3, third quartile; BOP, bleeding on probing; PCR, plaque control record;

PD, probing depth

At T_10,_ the median probing depth value was 2.8 mm (Q1: 2.5 mm, Q3: 3.3 mm) in group AST and 2.7 mm (Q1: 2.6 mm, Q3: 3.2 mm) in group STM (intergroup *p =* 0.912). The median BOP values amounted to 0.0 (Q1: 0.0, Q3: 0.2) in group AST and to 0.2 (Q1: 0.0, Q3: 0.2) in group STM (intergroup *p =* *0.255*). For PCR, the median value at T_10_ amounted to 0.0 (Q1: 0.0, Q3: 0.2) in group AST and to 0.0 (Q1: 0.0, Q3: 0.2) in group STM (intergroup *p =* 0.890).

## Discussion

The present long-term randomized controlled clinical trial comparing two types of dental implants with non-matching implant-abutment junctions supporting fixed restorations at 10 years of loading revealed: i) high survival rates for both types of dental implants, ii) stable marginal bone levels, iii) a higher rate of technical complications in group AST, iv) a higher rate of peri-implant mucositis in group STM.

The present study demonstrates an overall survival rate of 100% on the patient level and 89.7% for group AST and 96.8% for group STM on the implant level. This finding is consistent with prior publications, which have also reported high implant survival rates in short- and long-term observation periods [1, 2, 15, 23–27].

The available evidence shows that both systems assessed in this study exhibited implant survival rates ranging from 90.9% to 100% (AST) and 96.5% to 99.3% (STM) during observation periods spanning 1 to 10 years [28–37]*.*

When utilizing two-piece dental implants featuring a non-matching implant-abutment junction, studies report the maintenance of marginal bone in close proximity to the implant shoulder [14, 38–41].

The present investigation reveals that at the 10-year time-point, the median changes of the marginal bone levels between baseline and 10 years amounted to a loss of 0.07 mm for group AST, and to a gain of 0.37 mm for group STM. While the intergroup difference rendered a statistical significance, the clinical significance of this disparity remains uncertain. Absolute marginal bone levels between the two implant systems were clinically negligible at 10 years (median difference of 0.26 mm). Nevertheless, this difference is in line with recent publications. In a recent meta-analysis comparing the same systems amongst others, present study’s group AST showed favorable marginal bone maintenance. The authors also pointed out that even if statistically significant, the mean amount of marginal bone level change is small (MBL change ATO: -0.29 mm; SLA: -0.83 mm; NBT: -0.87 mm) [24].

The rates of technical complications differed between the two implant systems investigated, with group AST demonstrating more technical complications (27.0%) than group STM (15.6%) during the observation period of 10 years overall. Interestingly most of those complications occurred at earlier time-points, leaving rates of only 8.7% (AST) and 0% (STM) within the last two years of follow-ups (between T_8_ and T_10_). Minor chipping was the predominantly observed complication, followed by screw loosening. Apart from one abutment fracture in group AST, all complications could be treated chair side with a minimal investment in time and cost.

Comparing those rates to priorly published literature, they are higher than the reported range between 5 and 15% over 5 years for implant supported single crowns [2] and 4.6% for FDPs after 5 and 19.9% after 10 years respectively [42]

However, looking at the overall annual complication rate for implant supported single crowns in a systematic review and meta-analysis (4.2%, ranging from 1.7% to 15.5%), the results are in line with the technical complication rates withing the last two years of follow-ups in present study (8.7% for group AST and 0% for group STM) [15].

A recently published 6-year retrospective study investigated screw loosening with respect to several parameters, showing an overall rate of 7.2%. The highest frequency of screw loosening was investigated in the first 6 months after loading, predominantly in the molar region and significantly more frequent in implants with external than internal implant-abutment connections [43].

The higher rates of technical complications in present study can be attributed to a difficult comparison with prior literature. In a systematic review of outcome and measures, Sailer et al. state, that additionally to the finding that proshtetic failure is the least reported outcome measure, an inconsistent reporting about chippings and extended fractures of the veneering ceramic was found [7]. Small chippings being the predominantly observed complication in present study, it might explain the incongruence with prior literature, regarding technical complications.

In the current investigation, the incidence of peri-implant mucositis was found to be 29.7% in group AST and 50.1% in group STM. The prevalence of peri-implantitis was 0% in group AST and 6.3% in group STM, aligning well with the conclusions of recent systematic reviews.

Previous systematic reviews have investigated the prevalence of peri-implant mucositis and peri-implantitis ranges between 19% – 65% and 1% to 47% respectively [44–47]. The observed minimal occurrence of peri-implantitis can be attributed to a personalized maintenance protocol and effective oral hygiene practices that were consistently maintained by the majority of the subjects throughout the entire observation duration. (PCR at baseline: 0.1 and 0.2 at 10-year FU in group STM and 0.1 in group AST at both time-points). Most of the data assessing prevalence of peri-implant mucositis and peri-implantitis is sourced from studies that employ observational methodologies. This could account for the variations in the frequency of biological complications when compared to the present investigation, which is of a prospective design. It is noteworthy to mention, however, that the definition of peri-implant mucositis and peri-implantitis varies in the scientific literature and has not been consistently reported according to the newest classification, the consensus report of the 2017 World Workshop on the Classification of Periodontal and Peri‐Implant Diseases and Conditions [21, 22, 48].

One limitation of the present study is the absence of a formal sample size calculation. The primary outcome of interest at the time the study was initiated was the change in marginal bone levels, which was considered as a key parameter/metric for assessing implant success. Since data on STM was not available at the time, a convenience sample of patients was recruited based on the required number. Despite this limitation, the results of this study are deemed to be representative of a general dental practice, as a wide range of inclusion criteria were employed, limited only to fixed restorations. However, the wide range of inclusion criteria, which encompassed various factors such as implant location (maxilla, mandible, anterior, posterior), the use of guided bone regeneration (GBR), type of healing (submerged, transmucosal), loading time, retention type, and restoration material, may also be considered a potential drawback. The vertical component of the prosthetic part, mucosal thickness, and the anatomic location of the implants are recognized as crucial parameters that exert influence over peri-implant tissue stability. While these aspects were not reported within the scope of this study, their potential impact remains relevant for further investigation.

## Conclusion

The study revealed that the survival rates after 10 years were high irrespective of the implant system used. Marginal bone levels were observed to be in close proximity to the implant shoulder, with minimal changes noted over a 10-year period. Differences calculated for MBL changes were statistically significant in favor of STM, but considered to be clinically negligible. Technical complications were more frequently encountered in group AST, while group STM had a higher prevalence of peri-implant mucositis.

## Data Availability

All data supporting the present investigation is available upon request.
